# Dental Calculus Deposition: Correlation With Salivary Statherin and Calcium Levels

**DOI:** 10.1155/ijod/5356016

**Published:** 2026-01-07

**Authors:** Pranjali Hase, Vandana Shah, Shilpa Gunjal, Bhari Sharanesha Manjunatha, Deepak Gowda Sadashivappa Pateel

**Affiliations:** ^1^ Department of Oral and Maxillofacial Pathology, K M Shah Dental College and Hospital, Sumandeep Vidyapeeth, Vadodara, 391760, Gujarat, India, sumandeepuniversity.co.in; ^2^ Division of Clinical Oral Health Sciences, School of Dentistry, IMU University, Bukit Jalil, 57000, Kuala Lumpur, Malaysia, imu.edu.my; ^3^ Basic Oral Medicine and Allied Dental Science, Faculty of Dentistry, Taif University, Al Hawiyah, Saudi Arabia, tu.edu.sa; ^4^ Department of Oral Pathology Oral Medicine, Faculty of Dentistry, Mahsa University, Bandar Saujana Putra, 42610, Selangor, Malaysia, mahsa.edu.my

**Keywords:** calcium, dental calculus, saliva, statherin

## Abstract

**Objectives:**

Salivary constituents have a wide range of functions, including oral calcium homeostasis. Salivary proteins, such as statherin inhibit the crystal growth of calcium phosphate in supersaturated solutions and interact with several oral bacteria to adsorb on hydroxyapatite. Concurrently, saliva, which is supersaturated with calcium phosphates, is the driving force for plaque mineralization and calculus formation. The present study has, thus, been carried out to correlate salivary statherin and calcium concentration with dental calculus formation.

**Materials and Methods:**

The study comprised 90 participants (58 males and 32 females) aged 20–40 years. Participants were grouped according to the calculus index viz. Group I (control calculus index), Group II (low calculus index), and Group III (high calculus index). After collecting saliva, the salivary calcium and statherin levels were estimated for each participant, and the data were entered into a master chart.

**Results:**

The mean salivary statherin levels were found to be 1.305 ± SD 1.302, 0.986 ± SD 0.591, and 1.21 ± SD 0.473 in Groups I, II and III, respectively. The calcium levels increased from Group I to Group III (2.221, 5.067, and 10.072 mg/dL, respectively). Salivary calcium levels showed a significant positive correlation with the calculus index (0.639) (*p* < 0.001).

**Conclusion:**

Salivary concentrations of calcium appear to play an essential role in the dental calculus formation. Salivary statherin exhibited a statistically nonsignificant, modest negative correlation with both calcium concentrations and the development of calculus.

## 1. Introduction

The oral cavity is a moist environment, kept moist by saliva, which is essential for maintaining lifelong oral health [1]. Nature has endowed saliva with numerous functional capabilities that play a crucial role in maintaining the well‐being of the oral cavity, including mastication, deglutition, lubrication, buffering, defense against infections, prevention of tooth demineralization, and promotion of tooth remineralization [2]. Saliva is a complex fluid with a mixture of inorganic and organic constituents. The inorganic component of saliva comprises mainly calcium and phosphate salts [3].

The average calcium concentration in unstimulated whole mixed saliva is 4–6 mg/dL. A significant fraction of salivary calcium is diffusible and ionic, while the rest is in a bound form, mainly with the salivary proteins. In an alkaline environment, calcium aids in the remineralization of teeth through the formation of hydroxyapatite crystals, while in an acidic environment, it helps prevent the dissolution of enamel [4].

Dental calculus represents mineralized bacterial plaque and is composed primarily of calcium carbonate and phosphate, mixed with food debris and desquamated epithelial cells. Saliva supersaturation with calcium phosphate salts is the driving force behind the mineralization of bacterial plaque, leading to supragingival and subgingival calculus, as well as sialolith formation [5, 6]. The formation of calculus is a complex process similar to the remineralization of enamel [7]. However, this does not happen in every individual. The inter‐individual variation could be due to variations in the salivary calcium concentration and the salivary proteins, like statherin, involved in the calcium homeostasis [8].

Statherin, a small 43‐residue phosphoprotein relatively rich in tyrosine and proline found in human parotid and submandibular saliva, can inhibit the crystal growth of calcium phosphate salts and the spontaneous precipitation of calcium phosphate salts from their supersaturated solutions [8]. It has been proposed that statherin, shows high affinities for hydroxyapatite and inhibits unwanted and harmful precipitation of calcium phosphate salts in salivary glands and on the tooth surface. Thus, a protective, reparative, and stable environment is provided for the teeth [4, 5, 9, 10].

In vitro studies have revealed that the calcium and statherin enriched layer formed on the tooth surface may be associated with protective functions on the tooth surface [3]. A cross‐sectional study revealed a negative correlation of salivary calcium levels with salivary statherin [11]. The preliminary evidence available is linked to variations in sampling methodology and data analysis, and it requires further studies for a clear understanding of the complex oral environment. Hence, the present study was conducted to estimate and correlate salivary statherin and calcium levels in patients with varying degrees of dental calculus formation.

## 2. Methodology

The cross‐sectional study was approved by the Institutional Ethics Committee at Sumandeep Vidyapeeth University, Gujarat (SVIEC/ON/DENT/BNPG13/D14241). All the subjects were included after the written informed consent was obtained. The sample size for the study was calculated to be 85 samples using Fisher’s z‐transformation for correlation formula with *r* = 0.3, two‐sided *α* = 0.05, and power (1 − *β*) = 0.8 constants. A total of 90 participants were included in the present study.

The subjects, who were healthy and had various grades of dental calculus formation, aged between 20 and 40 years, were included in the present study. Participants with systemic diseases, such as diabetes mellitus, pathologies of the salivary, thyroid, and parathyroid glands, altered vitamin D metabolism, hormonal disturbances affecting calcium metabolism, or those on *β*‐adrenolytic drugs were excluded from the study.

After obtaining a brief dental history, the demographic details and oral examination findings were recorded for each participant. The amount of calculus formation was recorded using the Oral Hygiene Index–Simplified (OHI‐S) [12]. The participants were divided into three groups based on the calculus scores. No calculus present (calculus score 0) as Group I (control), supragingival calculus covering upto 1/3 of the exposed tooth surface (calculus score 1) and 1/3–2/3 of the exposed tooth surface (calculus score 2) as Group II (low calculus index), and supragingival calculus covering more than 2/3 of the exposed tooth surface (calculus score 3) as Group III (high calculus index).

### 2.1. Saliva Collection

The saliva was collected from all the individuals during the morning clinical session from 9 a.m. to 11 a.m. Each participant was asked to rinse the mouth, and unstimulated saliva was collected in sterile plastic tubes by the spitting method. The collected saliva was centrifuged at 4000 rpm using a bench centrifuge. The supernatant was collected and stored at −10°C immediately, and later, before 5 p.m., the collected saliva samples were moved to deep freezer storage at −70°C until further analysis.

### 2.2. Laboratory Analysis

The statherin levels were measured using an ELISA Kit (Antibody and reagents from Santa Cruz, N‐16, sc‐ 28112). The degree of color production, based on the quantity of statherin present in the sample, was evaluated using an ELISA Reader. Calcium levels were estimated using ACCUCARE Calcium Kit (AS III, Arsenazo III method, Lab Care Diagnostics), and readings were noted on a Spectrophotometer (650 nm, CECIL 1021, England) Autoanalyzer.

### 2.3. Statistical Analysis

The data were analyzed using the SPSS statistical package (version 29.0 SPSS Inc., Chicago, IL, USA). Descriptive statistics were performed to evaluate the distribution and normality of the data. The normality of quantitative variables was assessed by the Kolmogorov–Smirnov test, which revealed that the data were not normally distributed (*p* < 0.05). The chi‐square test was applied to compare gender and age distribution across different groups. Kruskal–Wallis ANOVA was used to find a significant difference between groups for salivary statherin and salivary calcium levels. Spearman’s rho correlation was used to find the correlation between salivary statherin, calcium, and CI‐S. The significance level was set at *p* < 0.05.

## 3. Results

### 3.1. Demographic Characteristics

A total of 90 participants, 58 (64.4%) males and 32 (35.6%) females, participated in the present study. There was no statistically significant difference in the gender distribution between the groups as assessed by the chi‐square test (*p* = 0.093). The mean age across the three groups is approximately 30 years and was found to be statistically nonsignificant (*p* = 0.204) (Table 1).

**Table 1:** Demographic characteristics of study participants.

Variables	Group I (Control)	Group II (low CI)	Group III (high CI)	Total	*p*‐Value
Gender, *n* (%)					
Male	17 (51.5)	29 (76.3)	12 (63.2)	58 (64.4)	^∗^ *p* = 0.093
Female	16 (48.5)	9 (23.7)	7 (36.8)	32 (35.6)	
Total	33 (100)	38 (100)	19 (100)	—	—
Age (years)					
Mean ± SD	31.52 ± 6.295	28.71 ± 5.713	30.16 ± 5.937	—	—
Minimum	20	20	22	—	—
Maximum	40	40	40	—	—
20–30	14	24	11	—	^∗^ *p* = 0.204
31–40	19	14	8	—	

^∗^ *p* < 0.05 indicates significant.

### 3.2. Independent Variables

The mean and standard deviation for salivary statherin (µg/10 µL) and salivary calcium (mg/10 µL) levels are depicted in Table 2. The mean statherin level in the healthy group (Group I) was 1.305 µg/10 µL. Whereas it was found to be 0.986 and 1.218 µg/10 µL, respectively, in Group II and III, as analyzed by Kruskal–Wallis ANOVA (Table 2).

**Table 2:** Comparison of salivary calcium and statherin levels in different calculus scores.

Groups	*N*	Mean salivary calcium (mg/dL)	Mean salivary statherin (µg/10 µL)
Group I (Control)	33	2.221 ± 1.991	1.305 ± 1.302
Group II (low CI)	38	5.067 ± 3.761	0.986 ± 0.591
Group III (high CI)	19	10.072 ± 4.795	1.21 ± 0.473
^a^ *p*‐Value	—	*p* < 0.001	*p* > 0.05

^a^Kruskal–Wallis ANOVA.

The salivary calcium levels increased progressively from Group I, Group II, and Group III with mean values of 2.221, 5.067, and 10.072 mg/dL, respectively, and the difference between the groups was found to be statistically significant (*p* < 0.001) when analyzed by Kruskal–Wallis ANOVA (Table 2). This significant increase in saliva calcium levels was found to have a positive correlation (0.639) with the overall mean calculus‐index score, which was found to be statistically significant (*p* = 0.001) as assessed by Spearman’s correlation test (Table 3). The present study also revealed a negative correlation between salivary statherin and salivary calcium levels (*r* = −0.024), which was found to be statistically not significant (*p* = 0.820) (Table 3).

**Table 3:** Correlation between salivary statherin level, salivary calcium level, and calculus score.

Variables	Calcium (mg/dL)	Statherin (µg/10 µL)	Mean calculus index
Calcium level			
Spearman’s rho	1.000	−0.024	0.639
* p*‐Value	—	0.820	<0.001 ^∗^
Statherin level			
Spearman’s rho	−0.024	1.000	−0.005
* p*‐Value	0.820	—	0.960

^∗^ *p* < 0.001 statistically significant.

## 4. Discussion

Dental calculus is preceded by plaque formation from the acquired enamel pellicle and primarily comprises mineral and organic components. Calcium and phosphate ions derived from saliva and crevicular fluid are subsequently adsorbed onto the plaque surface, culminating in the formation of dental calculus. The presence of specific membrane‐associated factors and the breakdown of nucleation inhibitors induce alterations in the supersaturation levels of calcium phosphate within the saliva, which ultimately trigger the initial mineralization of plaque and associated bacteria [6]. Consequently, an elevated calcium concentration in saliva has been documented in individuals predisposed to calculus formation. Experiments have shown that statherin is critical in maintaining calcium phosphate homeostasis in the oral environment. Its essential functions are binding to early crystal nuclei to suppress the onset of calcium phosphate crystallization and adsorption onto nucleated crystals to inhibit their growth [13–15]. The present study examined the role of salivary factors associated with dental calculus formation.

Unstimulated whole saliva was collected from all participants for the quantification of salivary calcium and statherin concentrations. The clinical evaluation encompassed the analysis of supragingival calculus utilizing the OHI‐S. Based on the scores from the calculus index, subjects were stratified into appropriate groups for subsequent analysis. The present study focused exclusively on supragingival calculus, as an assessment of subgingival calculus could provoke gingival bleeding, resulting in potential contamination of saliva specimens. Furthermore, subgingival calculus is characterized by distinct etiological factors and localized environmental influences, which could introduce variability and obscure the correlation between salivary components and calculus development [6, 16].

In the current investigation, the average calcium concentrations exhibited a progressive increase in relation to the formation of dental calculus and demonstrated a positive correlation (*r* = 0.639). This relationship was determined to be statistically significant (*p* > 0.001), and the results align with findings from prior research. A positive correlation of calcium levels with dental calculus formation is also noted in many studies [17, 18]. The salivary calcium is primarily saturated in saliva to facilitate the remineralisation process on the tooth surface. However, when an environment is created, it will lead to calculus formation. So, it’s important to note the factors affecting calculus formation to understand it better.

In the present study, mean statherin levels were found to have a very weak negative correlation (*r* = −0.005) with the mean calculus levels. The lack of correlation may imply that numerous other factors discussed below also play an essential role in regulating statherin levels. Furthermore, salivary statherin is a multifunctional protein that performs various tasks, ranging from calcium phosphate precipitation to the facilitation of selective bacterial colonization [19, 20]. Statherin additionally regulates bacterial adhesion, a fundamental step in the development of dental plaque and calculus. Research indicates that it diminishes the adherence of Streptococcus mutants, a microorganism implicated in the formation of dental plaque, to hydroxyapatite substrates [21]. Furthermore, it is essential to note that PRPs, histatins, and cystatins share functional similarities and characteristics with statherin that contribute to their specific roles in oral health.

The results of the present study add valuable knowledge to the biomedical field, supporting clinical applications and preventive measures in dental calculus formation. Clinical trials have demonstrated that chemical additives to toothpaste can inhibit calculus deposition [22]. A statherin mimicking polymer, chemically synthesized in the laboratory, showed effective anticalculus formation properties [23].

The results of the current study require validation through investigations employing larger participant groups. The role of the unbound and protein‐bound forms of salivary calcium can be assessed separately. Future studies should include a thorough evaluation of other dental calculus and oral health parameters, such as the status of oral hygiene, prevalence of dental caries, smoking, incidence of erosion, and salivary pH in relation to levels of salivary statherin and calcium, which would yield a more profound understanding of their interconnections and clinical implications.

## 5. Conclusion

Salivary concentrations of calcium appear to play an essential role in the formation of dental calculus. The weak negative correlation between salivary calcium and statherin levels and calculus highlights the potential protective role of statherin against calculus formation, suggesting that statherin activity could be beneficial.

## Ethics Statement

This study was approved by the Research Management Centre, Sumandeep Vidyapeeth, Vadodara SVIEC/ON/Dent/BNPG‐(3/1)14241.

## Disclosure

The authors take full responsibility for the content of the publication. All the authors have read and approved the final version of this manuscript.

## Conflicts of Interest

The authors declare no conflicts of interest.

## Author Contributions

Vandana Shah contributed to the conception, design, lab analysis, and interpretation, as well as drafted and critically revised the manuscript. Pranjali Hase and Bhari Sharanesha Manjunatha contributed to the lab analysis and methodology development. Shilpa Gunjal critically revised the manuscript and performed the statistical analysis. Deepak Gowda Sadashivappa Pateel contributed to the design, guided the research project, and critically revised the manuscript.

## Funding

The research was not funded by any agency or their university. The author, Dr. Vandana Shah, purely self‐funded it.

## Data Availability

The clinical and lab analysis records used in the present study are available from the corresponding author upon reasonable request.
