# Correction to “Dental Calculus Deposition: Correlation With Salivary Statherin and Calcium Levels”

**DOI:** 10.1155/ijod/9853817

**Published:** 2026-07-13

P. Hase, V. Shah, S. Gunjal, B. S. Manjunatha, and D. G. S. Pateel, “Dental Calculus Deposition: Correlation With Salivary Statherin and Calcium Levels,” *International Journal of Dentistry* 2026 (2026): 5356016, https://doi.org/10.1155/ijod/5356016.

In the abovementioned article, the correspondence details were incorrect. The correct information is shown below:

Correspondence should be addressed to Shilpa Gunjal; shilpagunjal@imu.edu.my and Deepak Gowda Sadashivappa Pateel; pateel@mahsa.edu.my

We apologize for this error.

